# Deployable Telescopic Tubular Mechanisms With a Steerable Tongue Depressor Towards Self-Administered Oral Swab

**DOI:** 10.3389/frobt.2021.612959

**Published:** 2021-03-05

**Authors:** Kirthika Senthil Kumar, Tuan Dung Nguyen, Manivannan Sivaperuman Kalairaj, Vishnu Mani Hema, Catherine Jiayi Cai, Hui Huang, Chwee Ming Lim, Hongliang Ren

**Affiliations:** ^1^ Department of Biomedical Engineering, Faculty of Engineering, National University of Singapore, Singapore; ^2^ Singapore Institute of Manufacturing Technology, A*STAR Singapore, Fusionopolis Two, Singapore; ^3^ Department of Mechanical Engineering, Faculty of Engineering, National University of Singapore, Singapore; ^4^ Department of Mechanical Engineering, National Institute of Technology, Tiruchirappalli, India; ^5^ Department of Otolaryngology-Head and Neck Surgery, Singapore General Hospital, Singapore; ^6^ Duke-NUS Graduate Medical School, Singapore; ^7^ Department of Electronic Engineering, Faculty of Engineering, Chinese University of Hong Kong, Hong Kong

**Keywords:** COVID-19, swab, self-administered, oropharangeal swab, low-cost, tongue depressor, stretchable sensors

## Abstract

Swabbing tests have proved to be an effective method of diagnosis for a wide range of diseases. Potential occupational health hazards and reliance on healthcare workers during traditional swabbing procedures can be mitigated by self-administered swabs. Hence, we report possible methods to apply closed kinematic chain theory to develop a self-administered viral swab to collect respiratory specimens. The proposed sensorized swab models utilizing hollow polypropylene tubes possess mechanical compliance, simple construction, and inexpensive components. In detail, the adaptation of the slider-crank mechanism combined with concepts of a deployable telescopic tubular mechanical system is explored through four different oral swab designs. A closed kinematic chain on suitable material to create a developable surface allows the translation of simple two-dimensional motion into more complex multi-dimensional motion. These foldable telescopic straws with multiple kirigami cuts minimize components involved in the system as the characteristics are built directly into the material. Further, it offers a possibility to include soft stretchable sensors for realtime performance monitoring. A variety of features were constructed and tested using the concepts above, including 1) tongue depressor and cough/gag reflex deflector; 2) changing the position and orientation of the oral swab when sample collection is in the process; 3) protective cover for the swabbing bud; 4) a combination of the features mentioned above.

## Introduction

With the spread of the COVID-19 pandemic throughout the world, mass testing of the population has proved to be an effective method to contain and control the disease. The testing of the patient involves the collection of respiratory specimens through two modes of swab tests – a nasopharyngeal swab and an oropharyngeal swab (Petruzzi et al., 2020). Between the two modes, the oropharyngeal is often preferred by patients as it causes less pain or discomfort (Wyllie et al., 2020). The procedure of oropharyngeal swab is as follows: 1) a tongue depressor used to depress the tongue, allowing examination of the throat and suppress gag reflex; 2) a swab directed towards the rear wall of the oropharynx near the tonsils and is rotated a few times before removal. Due to the complexity of hand-eye coordination in the swabbing process, the oropharyngeal swab is carried out mainly by healthcare workers at present. However, this practice limits the testing capacity based on access to swabs, workforce availability, increased rate of infection risks, psychological distress, and workload to healthcare workers (Greenberg et al., 2020; World Health Organization, 2020; Schwartz et al., 2020; Shechter et al., 2020).

Currently, the most common solution to counter this issue is to adopt a robotics system to replace healthcare workers during the swabbing process (Yang et al., 2020). This system prevents cross-infection and collects the patient's respiratory samples through automation or remote-controlled actuation (Hunt et al., 2008; Li et al., 2020; Robotics, 2020). These designs, while being novel and effective in a modern testing facility, are still rather resource-intensive for applications such as at-home sample collection or testing in remote, rural areas. Hence, it is important to develop a simple and effective self-administered oropharyngeal swab for the particular group of patients with the difficulty of oropharynx exposure.

Herein, we explore new designs of the oral swab equipped with stretchable sensors which are more suitable for self-administered compared to the rigid and inflexible traditional swabbing methods. At the same time, it is also simpler and less resource-intensive compared to a robot-assisted swab system. In particular, we are interested in swabs that are: 1) "cooperative" (i.e., safe to operate in contact with humans); 2) simple to construct, inexpensive (suitable for the single-use purpose); 3) intuitive to use (to be self-administered by patients); 4) effective (despite natural reaction such as cough, sneeze, and gag by the patient). Without resorting to complex systems, the simplest method to achieve cooperativity is to embed this characteristic straight into the material property of the swab by constructing them out of lightweight materials that are mechanically compliant to external forces (Hartenberg and Danavit, 1964; Gaiser et al., 2012). We also seek to achieve cooperativity by focusing on applying deployable telescopic tubular techniques to create a complaint mechanism with closed kinematic chains.

## Materials and Methods

### Actuation Mechanism

**FIGURE 1:**
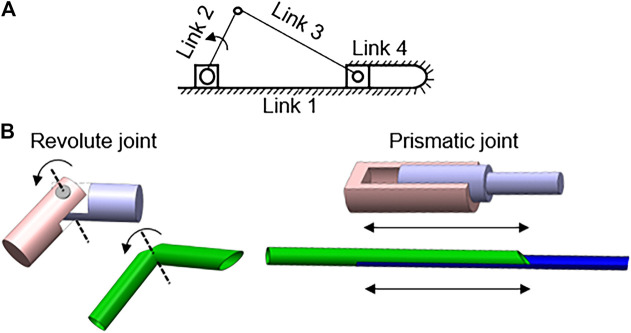
Combination of slider-crank linkage and kirigami-based deployable telescopic tubular mechanical designs **(A)** Variation of slider-crank mechanism with link 1 grounded; **(B)** Replication of joints on polypropylene tubes with simple cuts.

To develop a suitable design for an oral swab, deployable and foldable telescopic tubular mechanical designs were explored to fabricate a slider-crank linkage. A traditional in-line slider-crank mechanism consists of one sliding pair and three revolute pairs. It allows the translation of linear sliding motion to rotatory motion or vice versa (Nemiroski et al., 2017). In the proposed designs, an inversion of the closed slider-crank chain is utilized, similar to a reciprocating-engine mechanism except that link 1 is fixed (Figure 1A). The four links in this mechanism are connected by 3 revolute joints and 1 prismatic joint with 1 degree of freedom (DoF) each. Traditionally, slider-crank linkage comprises rigid bodies made from a hard material which is rather bulky and un-cooperative. To address this, we achieved a slider-crank mechanism by applying kirigami techniques to a soft material (polypropylene tube) to alter its material property directly. A kirigami-based mechanical system allows us to create sophisticated three-dimensional (3D) motion from a 2D surface (Felton et al., 2014). A kirigami-based compliant mechanism also has a compact mechanical footprint and volumes while having high cooperativity with humans (Nelson et al., 2019).

**FIGURE 2:**
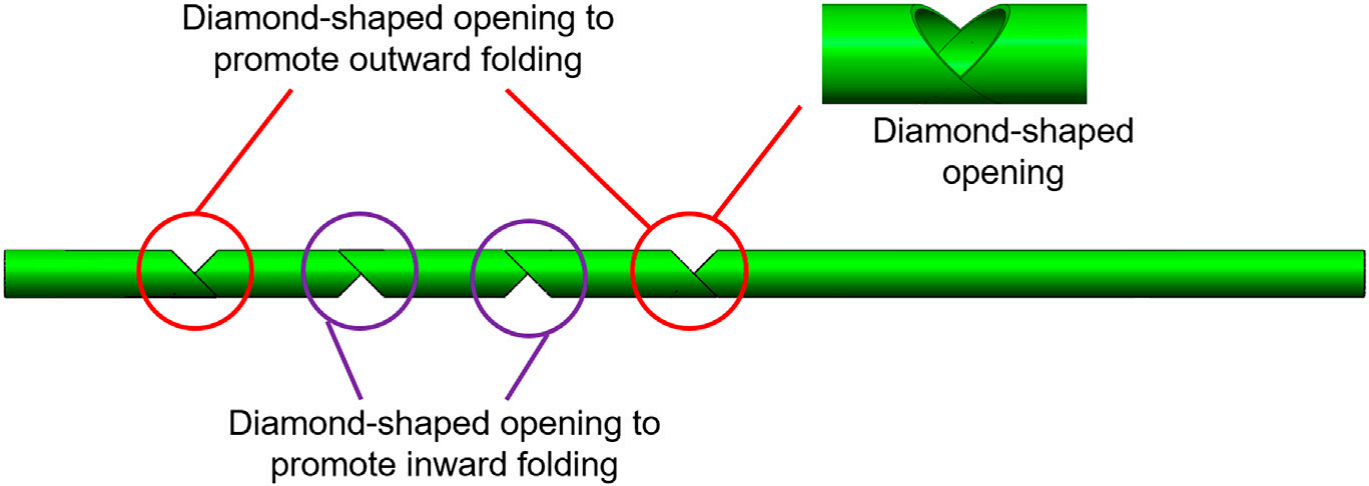
Detailed schematic of the design of the kirigami cuts to promote inward and outward folding.

To fabricate a compliant slider-crank linkage from deployable telescopic tubes, kirigami cuts of size ∼2.3–5 mm, are made to form the hinges/joints (Figure 1B). The type of cut determines the compliance of the bending motion. To ensure the motion of the links and joints follow the desired path, diamond shape opening has been cut to promote either inward or outward folding as seen in (Figure 2). Through experiments, it is realized that a curvilinear cut retains its structural rigidity ptduring bending motion, making it controlled and smooth. Also, the load-bearing capacity is higher with the curvilinear cut than a simple linear cut for joints. The continuum sliding pair is fabricated by inserting two straws of different diameters. Rigid links are formed using adhesive tapes. These slider-crank linkages are attractive as the basis for a new design of self-administered oral swab for three reasons; 1) easily accomplished joint/link to a structure by cutting a notch at the desired point of flexure, 2) complex actuation such as multi-directional or rotatory motion achieved by simple linear translational sliding, 3) placements of constraints through links and joints allowing precise control of equipment in operation.

### Straw Swab Designs

#### Bistable Swab With Tongue Depressor (BSTD)

**FIGURE 3:**
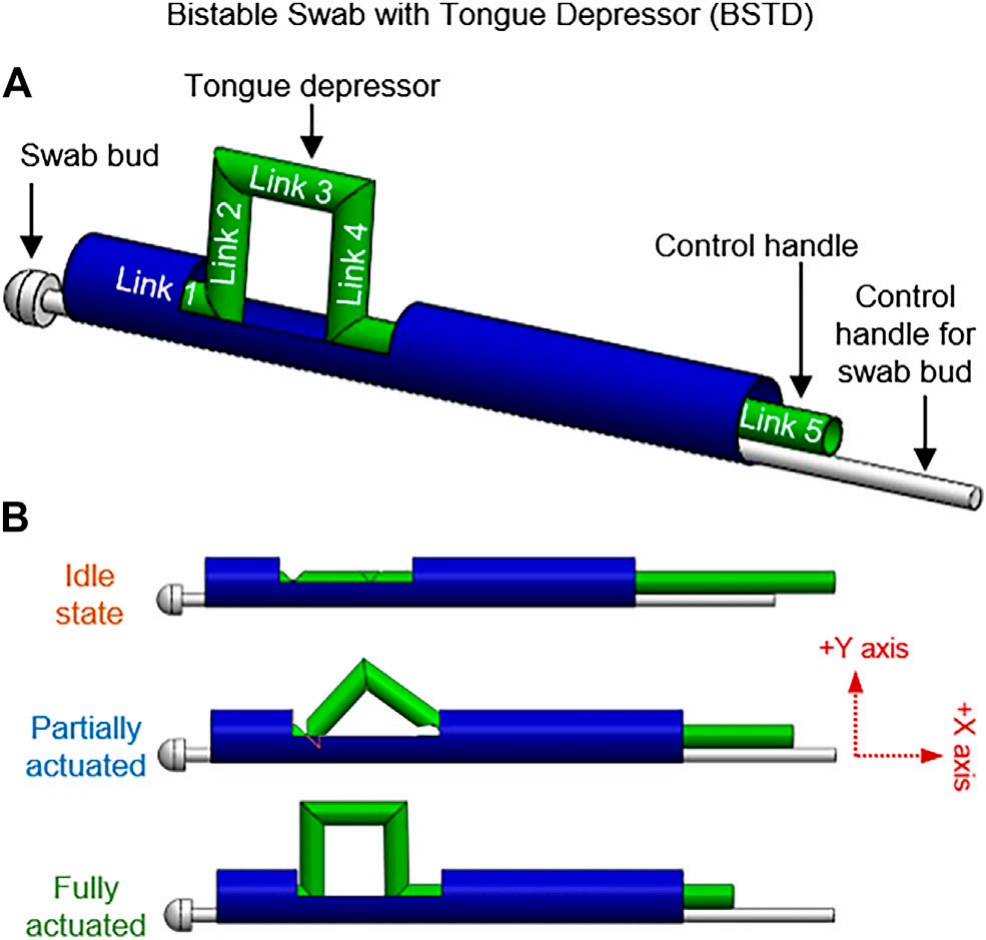
Bistable swab with tongue depressor (BSTD); **(A)** Annotations of the links and various components of the BSTD design, **(B)** BSTD at different stages of actuation.

Bistable swab with tongue depressor (BSTD) and its components are shown in Figure 3A. The construction of three revolute joints to a slider-crank mechanism allows developing a monostable swab with a tongue depressor. This design contains four linkages with 2 DoF. The Linear translation motion of link 4 deploys links 2 and 3 in the *Y*-axis. However, this structure has a possibility of retraction upon resultant force from the tongue. By adding an extra link and joint, a bistable system can be produced to create a planar closed kinematic chain. The closed kinematic chain has 5 links and 5 lower pairs, giving it 2 DoF. Linear translation motion of link 5 deploys link 3. By additional DoF to the system, link 3 deploys vertically (*Y*-axis) while remaining parallel to the main body axis. This allows link 3 to act as an effective tongue depressor and remains compact.

Figure 3B demonstrates the motions of the linkages from initiation to motion to finish. While in operation, BSTD can be placed in the patient mouth with the undeployed depressor facing downward toward the tongue. Once placed in the desired position, the depressor can be activated by sliding the control handle, causing the closed kinematic chain to pop down. Once activated in the patient mouth, the collection swab can be controlled with 2 DoF – sliding and rolling. Upon completion, the tongue depressor can be brought back to the idle state by retracting the control handle.

#### Side Swab Actuator (SSA)

**FIGURE 4:**
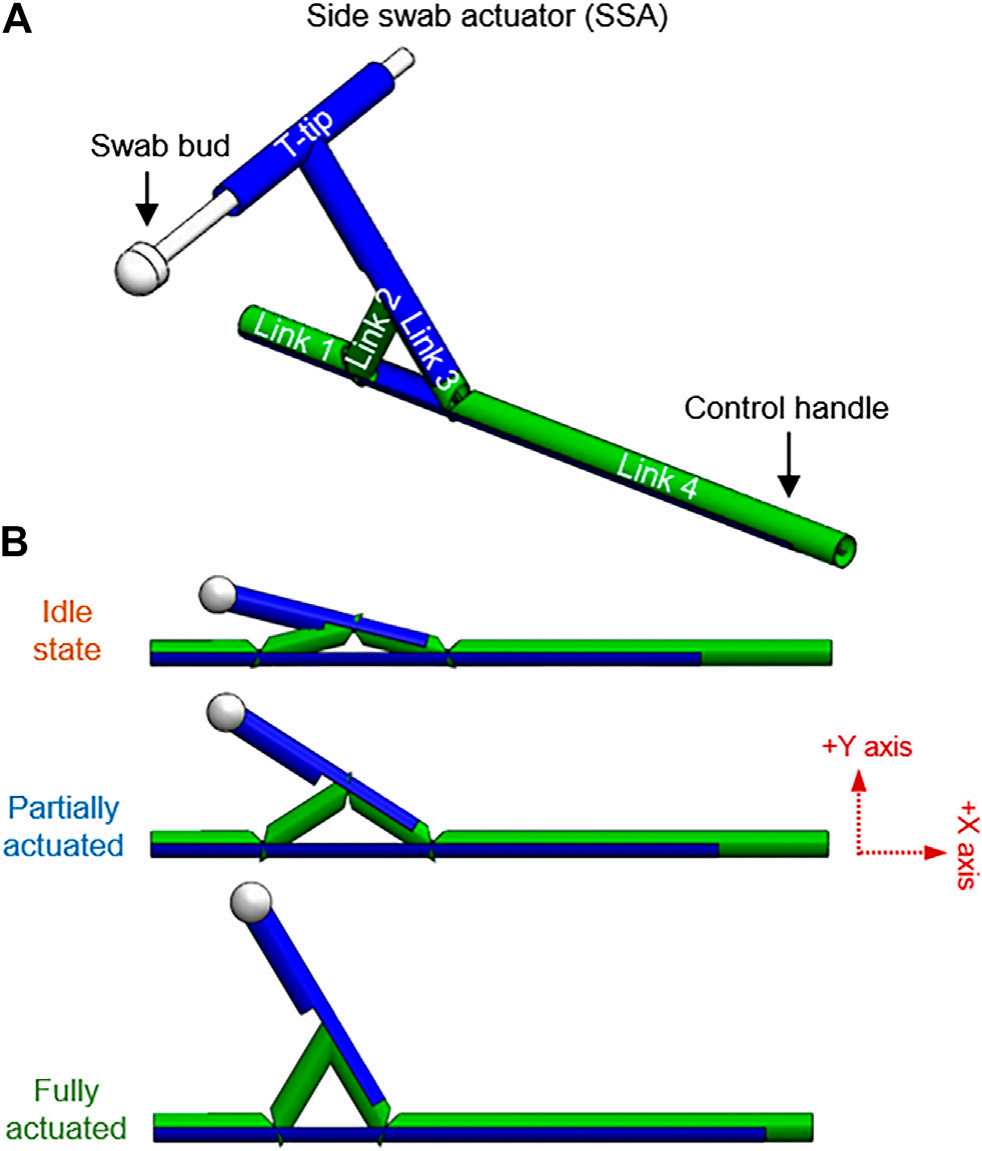
Side swab actuator (SSA); **(A)** Annotations of the links and various components of the SSA design, **(B)** SSA at different stages of actuation.

Side swab actuator (SSA) takes another approach where the closed kinematic chain is used to change the direction of the motion of the swabbing bud. Figure 4 demonstrates SSA at a different stage of its motion. Similar to the monostable design, three revolute joints are added to the slider-crank mechanism. Additionally, link 3 is extended with a T-tip to accommodate for the swab bud, normal to the slider. The linear sliding motion of link 4 is translated into rotating motion through the one DoF closed kinematic chain. This SSA design allows the patient to hold the swab perpendicularly to their mouth, instead of parallel. They are thus providing a more convenient hand position for the user. Also, this swab design, when operated by healthcare workers, allow them to stand facing away from the patients while still being able to collect the sample effectively.

#### Rotational Swab With Tongue Depressor (RSTD)

**FIGURE 5:**
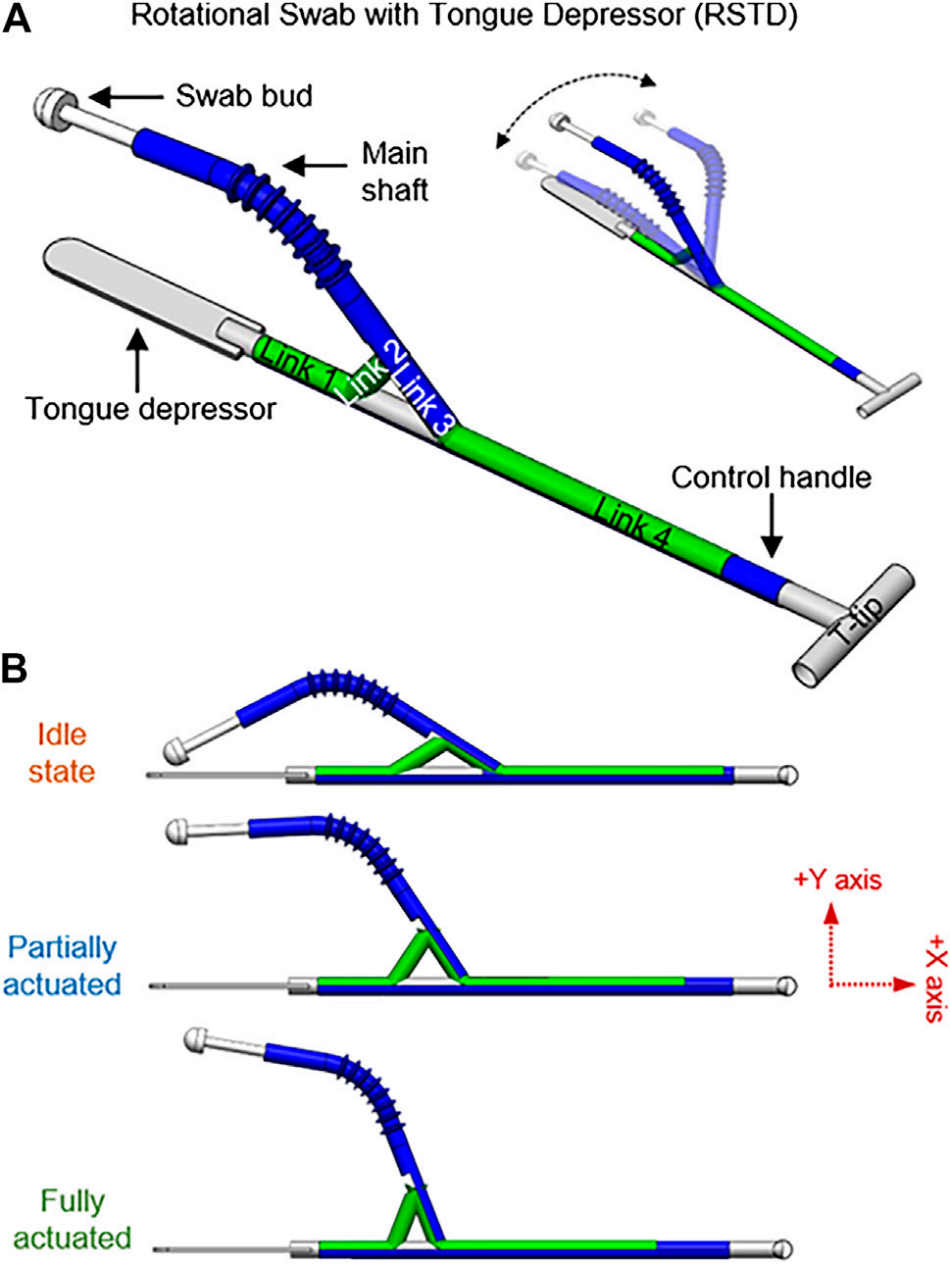
Rotational swab with tongue depressor (RSTD); **(A)** Annotations of the links, various components, and rotational function of the RSTD design, **(B)** RSTD at different stages of actuation.

Rotational swab with tongue depressor (RSTD) has four linkages, the main shaft to hold the swab bud, T-tip for rotational motion control, and a tongue depressor to minimize gag reflex (Figure 5). RSTD possesses 2 DoF; 1) sway in medial-lateral axis translated from the sliding motion of link 4 through the slider-crank linkage, 2) *X*-axis roll of the main shaft of the swab through the T-tip attached to link 4. During operation, this design allows the user to adjust the angle of the swab bud in the lateral axis through the slider. Once the desired angle is obtained, the swab bud can be rotated to facilitate the swabbing process.

#### Tendon-Driven Swab With Tongue Depressor (TSTD)

**FIGURE 6:**
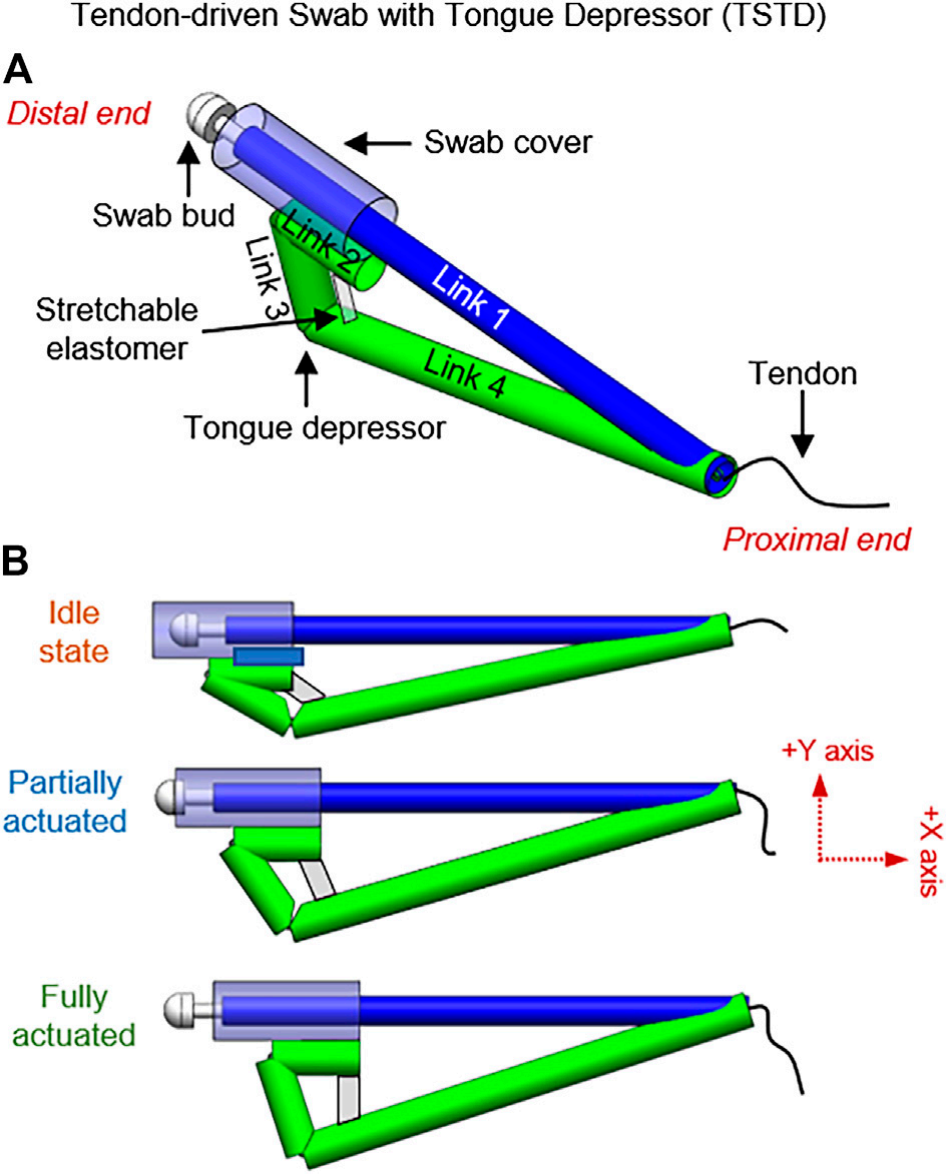
Tendon-driven swab with tongue depressor (TSTD); **(A)** Annotations of the links and various components of the TSTD design, **(B)** TSTD at different stages of actuation.

In the Tendon-driven Swab with Tongue Depressor (TSTD) design, the 1 DoF slider-crank linkage is equipped with four features: tongue depressor, swab cover, stretchable elastomer, and tendon at the proximal end. Figure 6 shows the swab at a relaxed state where the tongue depressor is deactivated, and the swab bud is enclosed in the swab cover. The swab device can be actuated by retracting the tendon that runs through links 2,3, and 4. Upon actuation, the swab moves linearly towards the proximal end, exposing the swab bud. This linear motion also activates links 3 and 4 to be deployed as a tongue depressor. After the sample is collected, the depressor is retracted, and the cover is closed simultaneously by applying pressure on link 4. Alternatively, the storage elasticity in the stretchable elastomer between links 2 and 4 allows automatic retraction.

### Sensorization

Self-administered swabs pose a potential for injuries as the user is not a trained professional like a healthcare worker. Besides, most of the swabs rely on direct vision to understand the placement or orientation of the swab. The actuation in a constrained environment thereby restricts the visualization of the swab. Others depend on their hand guidance to approximately sense the alignment of the swab. This efficiency of sensing is gained through experience and practice. However, in most cases, self-administered swabbing is performed for the first time by the user without prior experience. Hence, it is important to make self-administered swabs more intuitive and provide full information to the user during the process. This sensing information creates more awareness and provides a sense of control and confidence to the user. Therefore, a means of feedback would provide accurate identification of the deployment stage, improve the control, employ safety with better precision, and enhance the effectiveness of the swabbing process.

**FIGURE 7:**
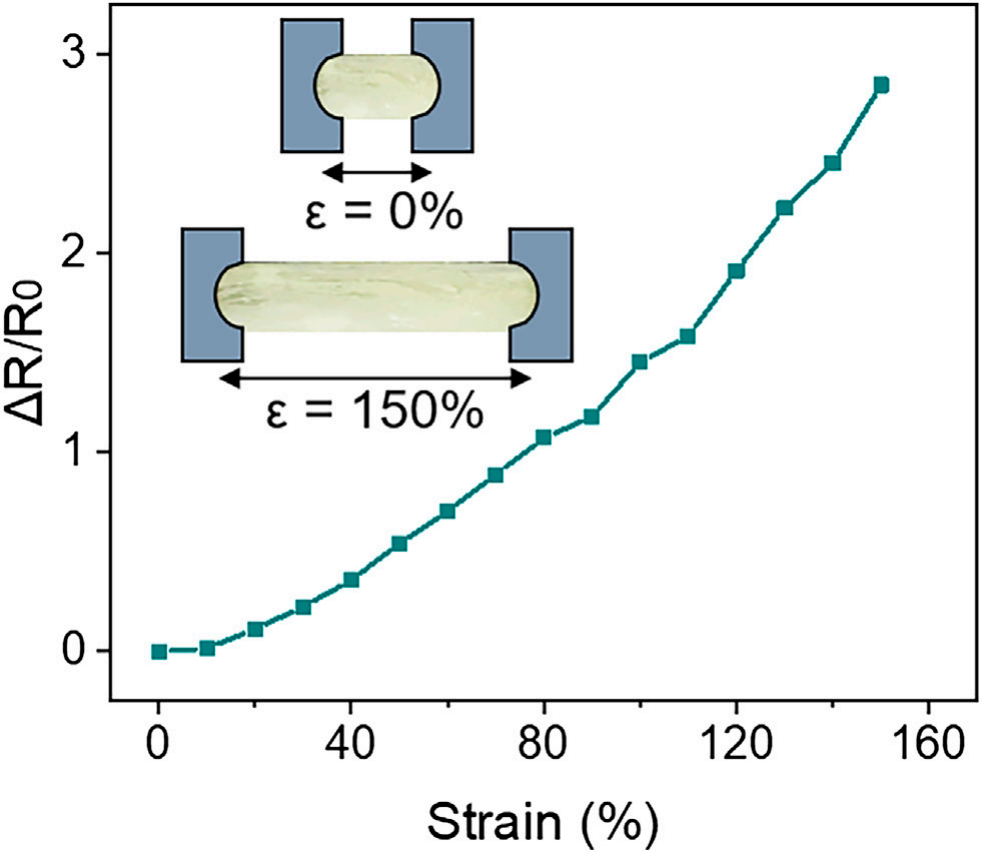
Strain profile of soft stretchable hydrogel-based strain sensor for strains up to 150%.

Directly mounting sensors on the self-administered swabs can help to evaluate their dynamic actuation. However, the sensors are required to possess good mechanical compliance that does not hinder the actuation mechanism. Hence, soft stretchable strain sensors hold the potential for this application. Hydrogel-based stretchable strain sensors possess good mechanical compliance, biocompatibility, and sensitivity. The sensor responds to tensile strains (ε) in the longitudinal direction by reflecting a change in electrical resistance(ΔR), acting on a piezoresistive principle. Its sensitivity can be represented through gauge factor (GF) where GF = (ΔR/R_0_)/ε, R_0_ is the initial resistance. The hydrogel-based strain sensor has a GF of 1.96 for strains up to 150%, as displayed in Figure 7.

Further, these sensors have the potential to behave as a restoring spring. The stored elastic energy upon stretching can be used as a medium of actuation, making them multi-purpose. This property is demonstrated in the TSTD design (Figure 6).

## Results

Experiments were conducted to understand the trajectory, complete workspace, amount of force required to actuate the swabs, force applied by the tongue depressor in each design, realtime performance monitoring using soft stretchable sensors, and the performance of the swabs on a human phantom.

### Motion and Workspace Analysis

**FIGURE 8:**
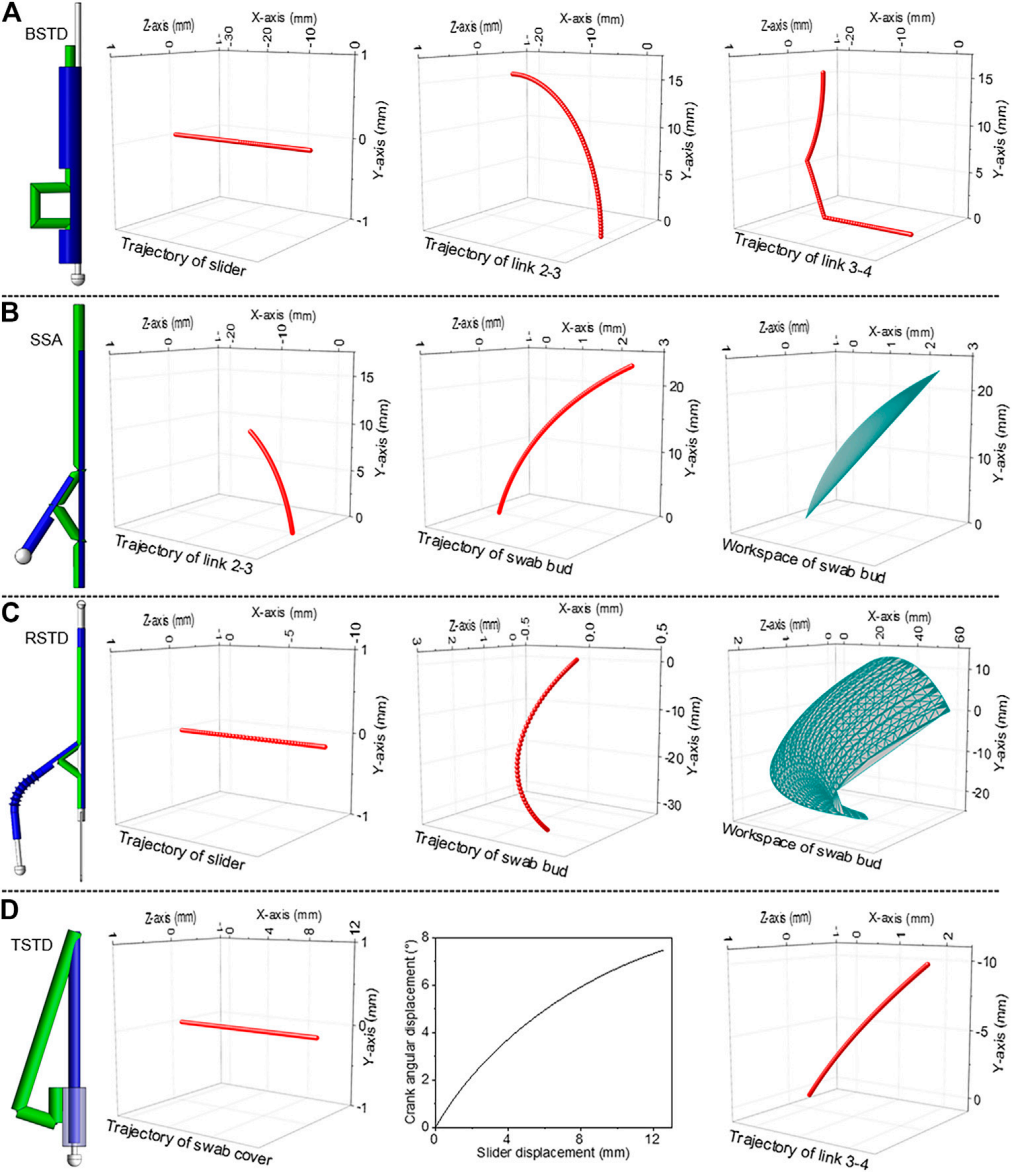
Trajectories and workspace analysis of the oral swab designs **(A)** BSTD, **(B)** SSA, **(C)** RSTD), **(D)** TSTD.

The behaviour of the swab designs is investigated using the simulation of the computer-aided design (CAD) models. The trajectories and workspace are analyzed for all 4 swab designs as the manipulators are driven to their limit positions (Figure 8). For the BSTD design, since link 3 is responsible for the gag deflecting feature, trajectories of the joint connected to it (link 2 and 4) are examined. The motion of link 5 (control handle-slider) is also recorded to compare the input linear sliding motion to the vertical output displacement of link 3. The trajectories in 3D space are shown in Figure 8A. Overall, it is observed that with an input of 31.2 mm linear sliding displacement in the –X-axis, a vertical displacement of 15.6 mm in the +*Y*-axis is achieved. For SSA design, the point of interest in motion analysis is at the tip of the swab bud and joint between link 2-3. The trajectories and workspace are recorded (Figure 8B) as the control handle is linearly displaced for 14 mm. Overall, an angular displacement of 17.7° in the +*Y*-axis is attained for the swab bud with the 14 mm linear displacement input.

To examine the movement and evaluate the workspace of the swab for RSTD design, motion analysis is performed (Figure 8C). A constraint is placed on the rotary motion to limit the roll axis to 90° (45° in the +*Z*-axis and – *Z*-axis) and a linear sliding distance of 11 mm. These boundaries are chosen to set a realistic range of motion for the 3D model based on the oral cavity. The trajectory of the slider and the swab bud are presented in Figure 8C. At extreme points, it is observed that an 11 mm linear displacement in the –X-axis of the slider results in a 14.7° angular displacement in the –Y-axis of the swab bud. The entire workspace of the swab bud is also displayed in Figure 8C. In the TSTD design, to understand the simultaneous motion between the tongue depressor and the swab cover, link 2 and the joint between link 1 and link 4 are focused for investigation. The trajectories and workspace of these two subjects can be seen in Figure 8D. It is observed that a 13 mm linear displacement of the swab cover in the + *X*-axis is translated to a 7.5-degree angular displacement between link 1 and link 4 in the –Y-axis, which is responsible for the actuation of the tongue depressor.

### Force Analysis

**FIGURE 9:**
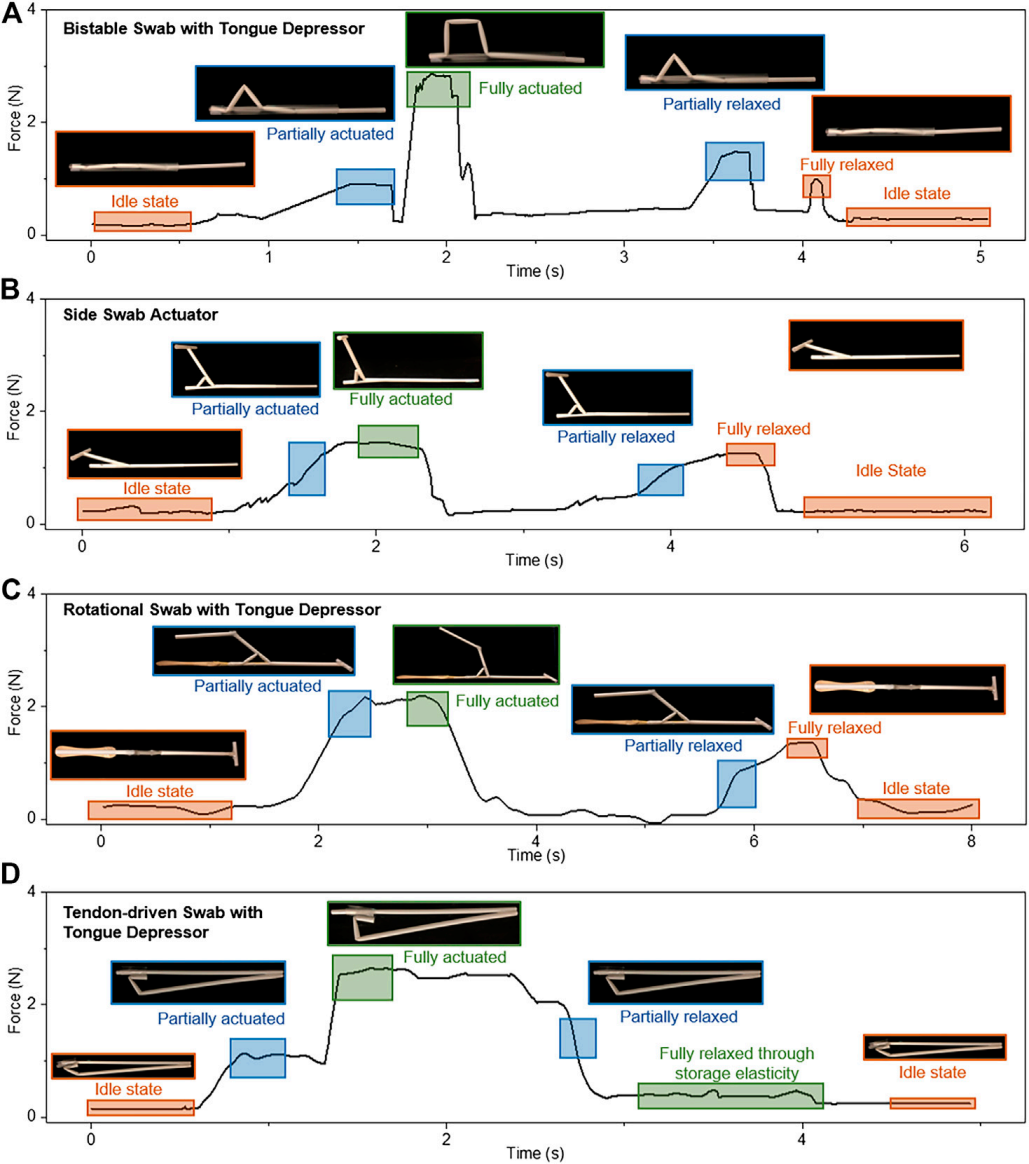
Force analysis of **(A)** BSTD, **(B)** SSA, **(C)** RSTD, **(D)** TSTD.

The force required to actuate the straw mechanisms to perform swabbing is measured. The applied force depends on the functional capability of the mechanisms, resistance between the polypropylene tubes, and the opposing force from the tongue depressor as it comes in contact with the tongue. The forces are measured using a semi-spherical 3axis force sensor (OMD-10-SE-10N, Optoforce Ltd.) with a sensing head (D = 10 mm) and resolution of 200mN. The sensor is placed in a plane normal to the swab models. For BSTD design (Figure 9A), an incremental force of 0.9N is required to initiate the actuation and land in a monostable position. A higher force ∼2.8N is required to deploy completely due to the resistance from the tongue on link 3.

Further, during the relaxation phase, two peaks occur as the process is done in two phases, monostable and bistable. The first peak occurs at 1.43N, and the second peak occurs while relaxing the slider-crank linkage at about 0.97N. Activating the mechanism on average requires much higher force than deactivating it.

The SSA design requires an average force of 1.35N to activate and deactivate the mechanism (Figure 9B). This value is similar to the maximum force required to initiate the slider-crank mechanism using the polypropylene tubes. Similar to BSTD, the RSTD design is operated with a constraint mimicking the tongue. The force profile gradually increases from the idle state to a fully actuated state, reaching a maximum value of 2.19N (Figure 9C), which is lower than the BSTD maximum force as the tongue depressor is isolated from the swab deploying mechanism. The force analysis for this design is done in 2 separate cases similar to design 1 due to the presence of a tongue depressor. When operating without external influence, the force profile gradually increases from the idle state to fully actuated until reaching a maximum value of 3N when the device is fully actuated. The second peak is received while relaxing the device at about 1.5N. It is observed that the force needed to relax the mechanism is approximately half of the force needed to actuate it. The retracting force is similar to the SSA design of ∼1.5N.

Further, the TSTD design creates a different force profile (Figure 9D). Force is applied for the deployment of the tongue depressor and removal of the swab cover simultaneously. During which, the stretchable elastomer stretches to its maximum. Summing to a maximum force of 2.64N, including the opposing force from the tongue constrain and the stored elasticity of the stretchable elastomer. This force has to be retained during the swabbing process, and a decline in the force allows automatic retraction due to the storage elasticity of the stretchable elastomer.

**FIGURE 10:**
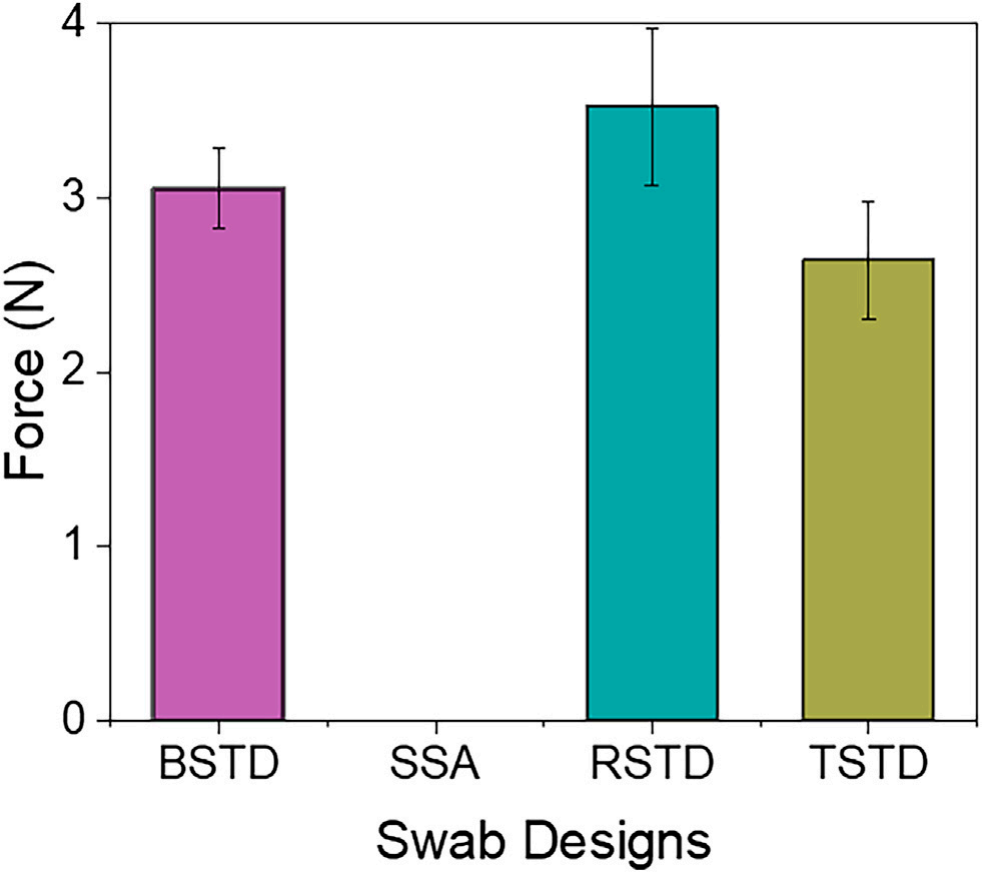
Force applied on tongue by the tongue depressor in the swab designs

The most conventional way to depress the tongue is by using a simple wooden flat stick (tongue depressor). When using the wooden tongue depressor, a common technique to prevent unconscious tongue slippage is by applying pressure on the tongue through the tip/frontal areas of the stick. Likewise, the tongue depressor proposed can be used. The amount of force/pressure applied on the tongue by the tongue depressor for each design is quantified using the semi-spherical 3axis force sensor (OMD-10-SE-10N, Optoforce Ltd.). The minimum and maximum force applied by the swabs is plotted in Figure 10. In most cases, the initial force of the tongue depressor is due to the deployment mechanism. However, the maximum force is still controlled by the user during the self-administered swabbing process. The maximum force applied by the BSTD design is 3.28N, whereas the RSTD design can apply 3.9N as the control is still with the user. The TSTD design applies a force of 2.98N upon deployment. It is essential to apply enough pressure to suppress the tongue. It is also advisable to avoid applying too much pressure, damaging the tissues {Ponraj, 2018 #22}. The amount of force that can be applied by the proposed tongue depressors are in the ranges of ∼2.9–3.9 N. Unconscious motion of the tongue will be restricted by this amount of force. Moreover, in the case of tongue slipping into the throat, the ultimate control lies with the user as it is self-administered swabbing. It is thereby enabling the user to stabilize the swab immediately.

### Realtime Performance Monitoring

**FIGURE 11:**
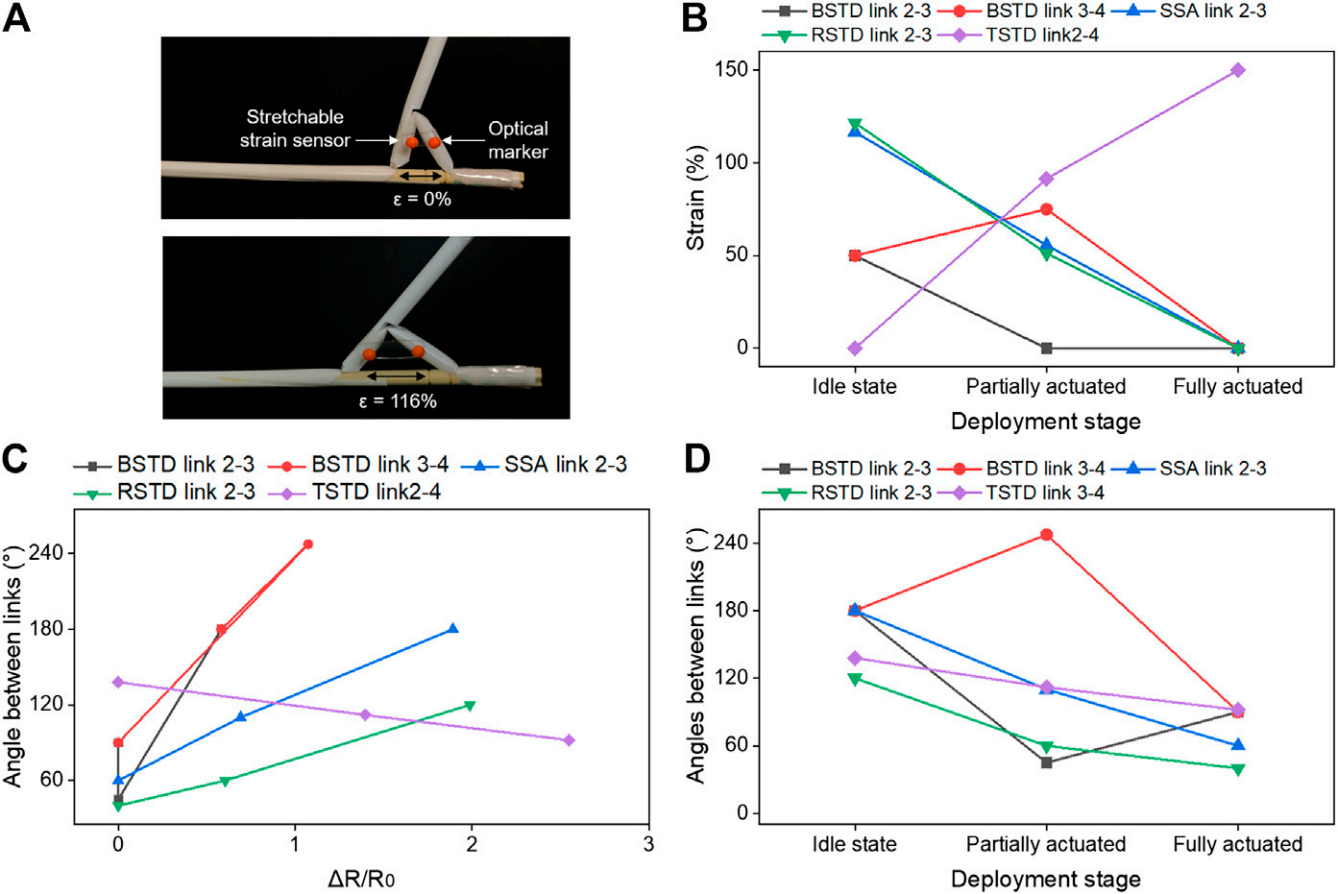
Realtime performance monitoring **(A)** Stretchable sensor placement on the swab design, **(B)** Strains experienced on sensors at various deployment stages, **(C)** ΔR/R_0_ response of the sensor and the determined angle between the links, **(D)** Angles formed between the links at various deployment stages.

The determination of strategic locations for sensor placement is by identifying the prominent links with maximum movements. For the BSTD, SSA, and RSTD design, the diagonal length between the links upon actuation is smaller than the point-to-point length in the idle state. Hence, the stretchable strain sensors are mounted at a fully actuated state. The RSTD design of the sensor is mounted between link 2, and link 3 is shown in Figure 11A. However, the initial distance between the link 2 and link 4 for the TSTD design is smaller. Hence the sensors are mounted in an idle state. The sensor's elastic storage property helps to relax the TSTD design from the fully actuated stage to the idle stage. The sensor response is exclusive to each swab design, which requires one-time calibration using the optical tracker before realtime monitoring for each swab design. The two orange points on the sensor are used to visually track the strains evolved to cross-validate the sensor performance with the angles. The optical tracking software (Tracker 5.0 (Douglas Brown^©^) is utilized for the same. The strains experienced by the stretchable sensor at various deployment stages are presented in Figure 11B. Further, the ΔR/R_0_ response of the sensor to various strains allows the determination of the change of angles between the mentioned links Figure 11C. Combining the data from the tracker software, strains of the sensors, and ΔR/R_0_ values, the deployment stages and angles between the links of the swab designs are determined in Figure 11D.

### Demonstration on Human Phantom

**FIGURE 12:**
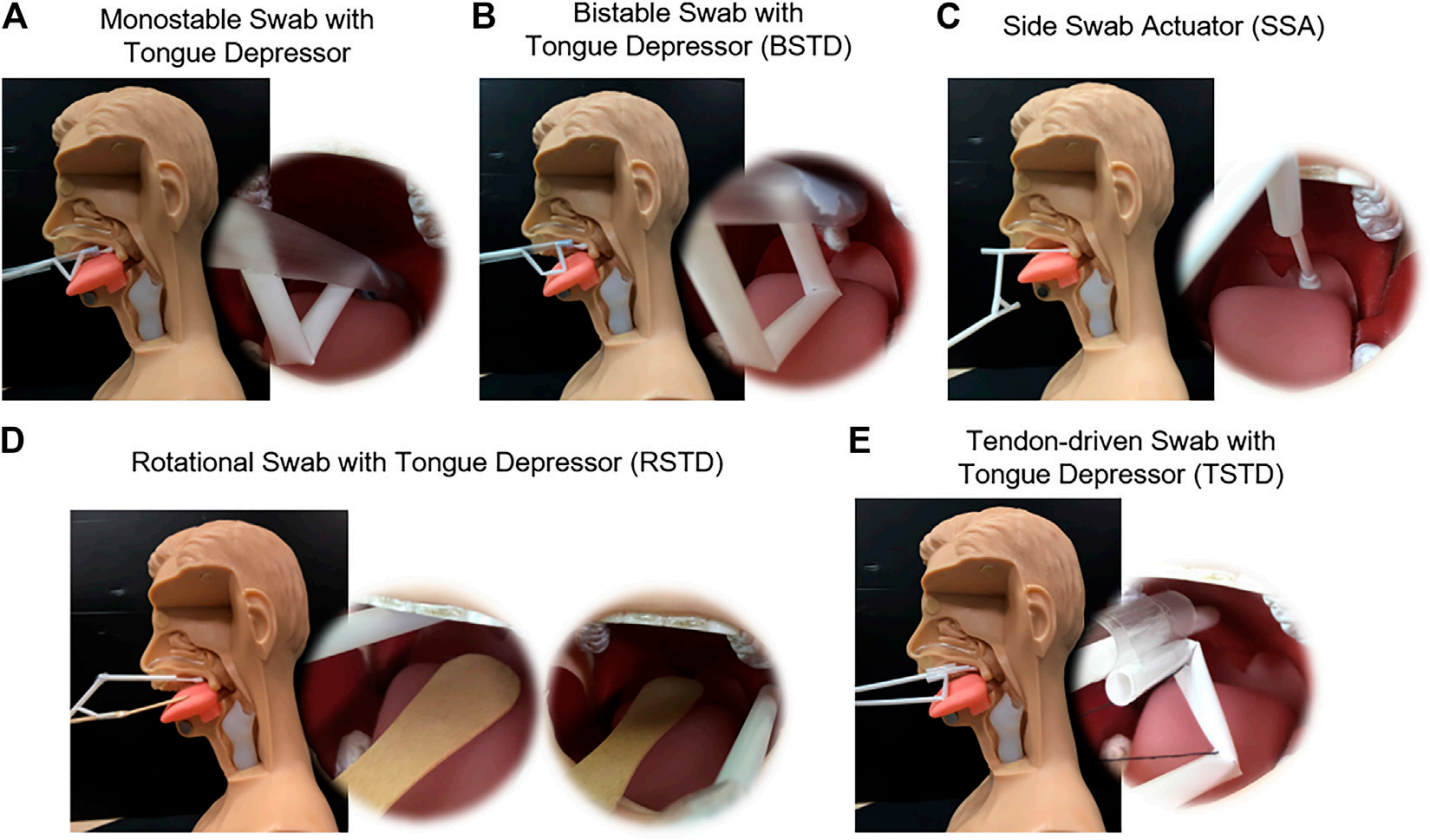
Demonstration of the proposed swab designs on the human phantom in the sagittal plane and endoscopic view **(A)** Monostable swab with a tongue depressor, **(B)** Bistable swab with tongue depressor (BSTD), **(C)** Side swab actuator (SSA), **(D)** Rotational swab with tongue depressor (RSTD), **(E)** Tendon-driven swab with tongue depressor (TSTD).

The self-administered swabbing process is demonstrated on a human phantom for all the four proposed swab designs, as shown in Figure 12. An endoscope (OVS1 Video System Portable Hysteroscopy System, Olive Medical Corporation) is mounted on the swab designs to validate the maximum reach of the swab towards the rear wall of the oropharynx near the tonsils. The effect of cough or sneeze during swabbing procedures are tested on the human phantom. During cough or sneeze, an impact is generated from the throat towards the oral opening and air is forcefully expelled. This impact applied an angular force on the swab which immediately reverts it to its more complaint partially actuated position.

## Discussion

A simple strategy to design and fabricate a sensorized self-administered oral swab using a closed-loop kinematic chain and kirigami-based deployable telescopic tubular structure is presented to address the potential occupational health hazard and reduce the workload to healthcare workers during the swabbing process. The central idea lies in the adaptation of the slider-crank mechanism to employ on a mechanically compliant structure. The combination of the tongue depressor with the swab aims to make this swabbing process compact, simpler, faster, and minimize gagging or choking of the patient. The four proposed designs demonstrate the opportunity to fulfil the three criteria set out for a self-administered swab 1) simple and inexpensive. They can be easily fabricated using readily available materials, suitable for the single-use nature of the oral swab. 2) designs equipped with sufficient features to offset potential hindrance such as natural response to oral swab and ensure the effectiveness of the collection of respiratory specimens. 3) cooperative designs (suitable to use with a human) made of soft materials. Additionally, the swab designs show the potential to be sensorized using soft stretchable strain sensors, allowing realtime monitoring of the swab performance and intuitive control. However, the future work is steered towards developing a golden ratio for the swab designs that could accommodate various dimension changes between the users. Also, the development of home-based swabbing kits could be a wise way to spend the resources.

## Data Availability

The original contributions presented in the study are included in the article/Supplementary Material, further inquiries can be directed to the corresponding author.
